# Polymer Nanostructuring for Flexible Lithium-Ion Batteries: A Systematic Review of Electromechanical Properties from TiO_2_ Nanocomposites to Semisolid Electrolytes

**DOI:** 10.1021/acsomega.5c12796

**Published:** 2026-06-03

**Authors:** Karine A. Kley, Guilherme L. Cordeiro, Lílian V. R. Beltrami, Clarissa Tolardo, Vitória S. Winck, Maicon Molon, Gislene Zehetmeyer

**Affiliations:** † Smart Materials, 644609 Instituto Hercílio Randon, IHR, 95181-899 Farroupilha, RS, Brazil; ‡ Electric Mobility, Instituto Hercílio Randon, IHR, 95181-899 Farroupilha, RS, Brazil; § Corrosion and Surface Protection Laboratory, LCOR, Programa de Pós-Graduação em Engenharia de Processos e Tecnologias, PGEPROTEC, 58802 Universidade de Caxias do Sul, 95070-560 Caxias do Sul, RS, Brazil

## Abstract

Nanocomposites are promising matrices for polymer electrolytes in lithium-polymer batteries, and TiO_2_ nanoparticles are widely explored to enhance dynamic-mechanical and ionic properties. However, despite increasing interest in epoxy-based electrolytes, no study to date has concurrently reported dynamic-mechanical and electrochemical properties for the same epoxy-TiO_2_ formulations. This prevents establishing structure–property relationships and represents a critical evidence gap. A search (2014–2024) across ScienceDirect, Scopus, Web of Science, and Springer identified eleven studies on epoxy-TiO_2_ dynamic-mechanical behavior and five on polymer–particle electrolyte performance. Pearson’s *r*-test (5% significance) revealed a strong inverse correlation (*r* = −0.97) between Tan δ and Tg in epoxy nanocomposites containing 30–40 nm TiO_2_ at 2–15 wt %, indicating a trade-off between damping and strength. Yet, although the review was originally designed to investigate whether these viscoelastic trends relate to electrochemical performance in semisolid epoxy-based electrolytes, such a link could not be established: no included study reported mechanical and electrochemical data for the same epoxy formulation. Limited data and experimental variability further hindered broader comparisons, especially between nanocomposites and polymer electrolytes. Notably, all conductivity improvements identified in polymer–particle systems occurred exclusively in nonepoxy matrices and therefore do not constitute evidence that TiO_2_-induced mechanical enhancements in epoxy resins translate into electrochemical gains. Nonetheless, two studies showed simultaneous mechanical and ionic enhancements, suggesting that nanoparticle-induced structural rearrangements may facilitate Li^+^ transport. Overall, this review highlights the potential role of nanoscale ceramics as nanostructuring agents while underscoring the need for future studies that jointly evaluate mechanical and electrochemical properties in epoxy-based electrolytes.

## Graphical abstract

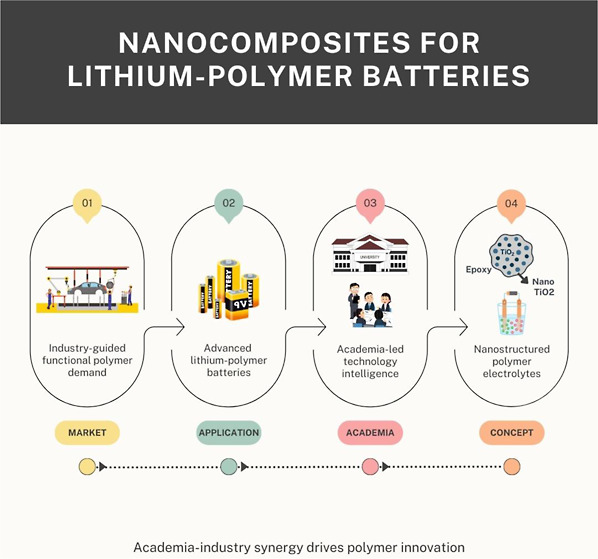

## 1 Introduction

Semisolid-state batteries are emerging as a pivotal electrochemical technology bridging the gap between liquid- and solid-state batteries, facilitating advancements in energy storage for transportation.^1^ Compared to conventional liquid electrolyte batteries, semisolid-state batteries with epoxy-based structural electrolytes offer numerous advantages, including enhanced ionic conductivity and improved chemical, mechanical, and thermal stability.^2–4^ While polymer-based electrolytes with superior mechanical properties have garnered significant attention,^2–4^ the research and development of semisolid-state technologies present a unique opportunity to accelerate electric mobility and decarbonize the transportation sector, particularly in Brazil.

As the eighth-largest automotive market in the world, Brazil has more than 1 million kilometers of highways within its vast road system. However, only 105 814 km of its road network are paved, with approximately 34% in fair condition and 16% in poor or very poor condition due to insufficient maintenance.^5,6^ This infrastructure scenario underscores the need for robust battery technologies that can withstand the challenging driving conditions.

In this context, the automotive industry faces a dual challenge: ensuring the mechanical robustness of battery systems under harsh road conditions while maintaining a high ionic conductivity for efficient energy delivery. These demands are particularly critical for semisolid-state batteries, which must perform reliably in vehicles exposed to vibrations, temperature fluctuations, and mechanical stress.^7,8^

From an industrial perspective—especially within automotive R&D environments—there is a growing interest in polymer electrolytes that can simultaneously offer structural integrity and electrochemical performance. This dual functionality is essential not only for extending battery lifespan but also for enabling safer and more efficient electric mobility solutions. Consequently, the development of TiO_2_-filled epoxy electrolytes emerges not merely as an academic endeavor but as a strategic response to a technological gap identified by industry stakeholders. As electric vehicles increasingly demand battery systems that are both mechanically resilient and electrochemically efficient, the dual enhancement of structural and ionic properties becomes indispensable.^7,8^

Recent industrial reports and academic research confirm this trend, highlighting the strategic importance of multifunctional polymer electrolytes in enabling high-performance, structurally integrated battery solutions. Yet, despite these promising developments, practical implementation faces significant hurdles. One such challenge is the supply of ceramic fillers—a key component in these systems—which represents another critical bottleneck. Materials such as garnet-type Li_7_La_3_Zr_2_O_12_, perovskite-type Li_0_._33_La_0_._57_TiO_3_, and sulfide-based fillers require controlled high-temperature synthesis steps, precise control of lithium volatility and strict stoichiometric accuracy to achieve the desired phase purity and ionic performance. While such processes are standard practice in specialized laboratory environments, large-scale production with adequate purity and reproducibility remains concentrated among a small number of suppliers, posing challenges in terms of scalability and cost. Furthermore, the production of nanoscale oxide and sulfide powders with ultralow contamination remains limited, further restricting the supply of these strategic materials.^9,10^

Against this backdrop, this study aims to bridge the gap between academic research and industrial application by systematically evaluating the role of TiO_2_ nanoparticles in enhancing both mechanical and ionic properties of epoxy-based electrolytes. By doing so, they contribute to the development of robust, multifunctional materials capable of meeting the evolving demands of electric mobility.

Significant progress has been made in enhancing the mechanical properties of epoxy-based electrolytes through the integration of additive-filled polymers, particularly those incorporating inorganic ceramic nanoparticles such as titanium dioxide (TiO_2_).^11,12^ Studies have consistently demonstrated that the inclusion of TiO_2_ nanoparticles improves the polymer‘s capacity to endure compressive or tensile loads.^11,12^ Li et al.,^11^ reported a 29% increase in compression strength with the addition of up to 10.0 wt % TiO_2_ (diameter: 7 nm) in a lithium-epoxy electrolyte. However, while nanoreinforcements enhance mechanical properties, semisolid-state batteries must also withstand the vibrations and stresses associated with driving on uneven roads. Thus, rigorous characterization of their mechanical properties under extreme operating conditions—such as fluctuating temperatures and cyclic stresses—is crucial. As illustrated in Figure 1, mechanical vibration, cyclic loading, temperature fluctuations, and microimpacts may induce deformation, interfacial debonding or local stiffness loss in the electrolyte, potentially interacting with broader failure pathways within the battery stack. Accordingly, rigorous evaluation of nanocomposite electrolytes under extreme or fluctuating operating conditions becomes essential. One approach to simulate these conditions involves using dynamic-mechanical analysis (DMA), where nanocomposites are evaluated through temperature- and frequency-dependent viscoelastic models.

**Figure 1:**
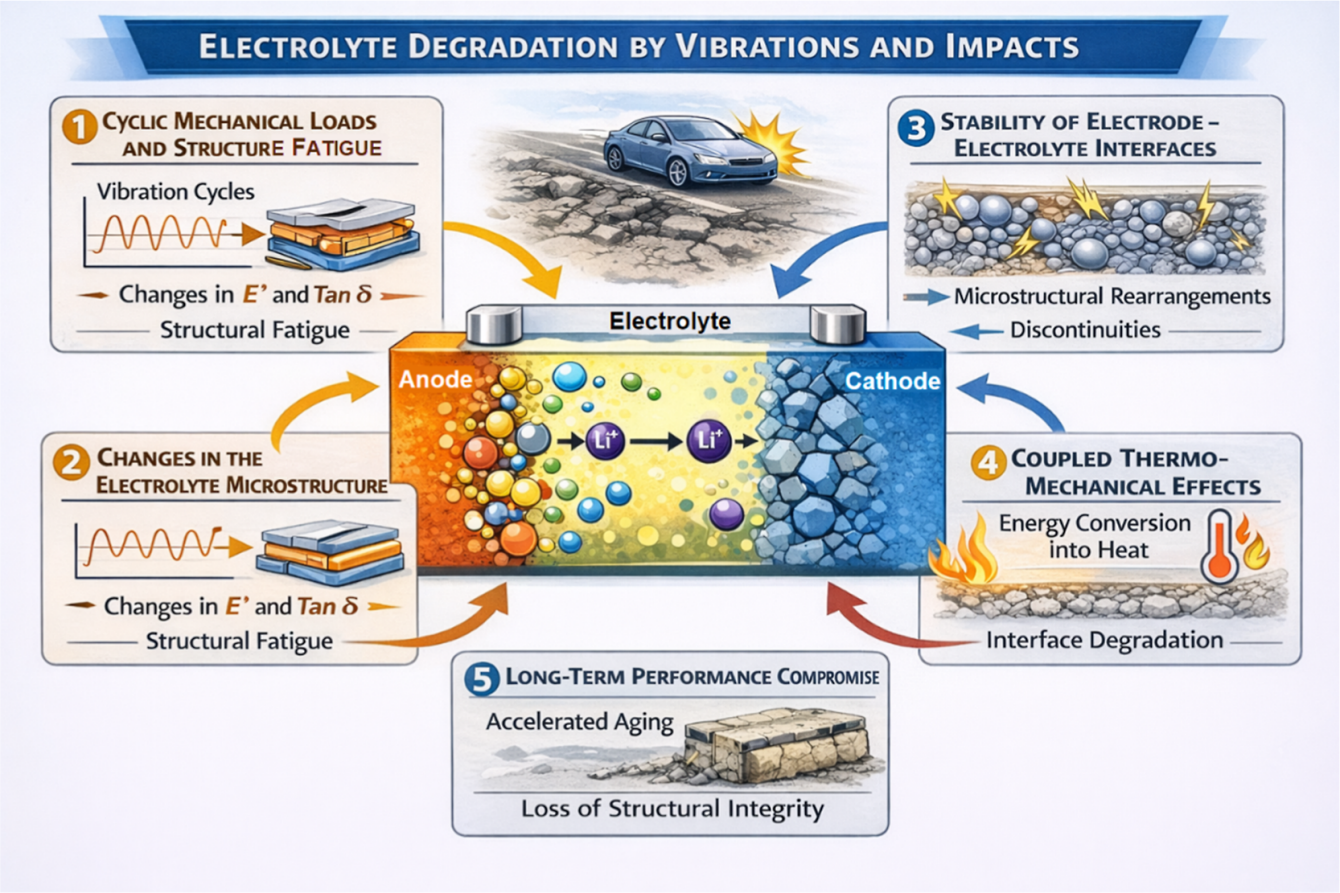
Schematic illustration of failure modes arising from vibration and cyclic loading, highlighting electrolyte deformation and instabilities at the electrode–electrolyte interface.^13–15^

Several studies^16–24^ have provided valuable insights into the viscoelastic behavior of nanocomposites. The cited studies mention that incorporating 1.0 wt % of TiO_2_ nanoparticles with diameters ranging from 15 to 35 nm significantly improves dynamic-mechanical properties. Nitesh et al.^20^ reported a 48% increase in elastic modulus for a hybrid composite containing 1.0 wt % TiO_2_. However, higher TiO_2_ concentrations, such as 10.0 wt %, often lead to reduced stiffness gains due to particle agglomeration and processing challenges, which create defects instead of reinforcing the structure. Goyat et al.,^21^ for example, observed only a 28% increase in stiffness with 10.0 wt % TiO_2_. This diminishing return is also evident in other properties, such as the loss modulus (*E*″), mechanical damping (Tan δ), and glass transition temperature (*T*_g_). On the other hand, only two studies^22,25^ used reduced concentrations of TiO_2_ (0.5 wt %) in their formulations and reported gains in their properties: Bakar et al.^22^ identified the highest modulus among all samples (M* = 465 MPa). Furthermore, an increase in *T*_g_, which may mean greater thermal stability of the material, indicating protective properties of the composite material with respect to the thermal insulation. Hiremath et al.^25^ observed that the addition of TiO_2_ nanoparticles improves the thermal stability of the sample. Moreover, they observed a marked improvement in the storage modulus. In general, TiO_2_ nanoparticles have more rigid characteristics than epoxy resin besides the large surface area of their small particles, which facilitates interaction with the polymer chain.^25^

Translating these improvements in the dynamic-mechanical properties of epoxy nanocomposites to the performance of TiO_2_-filled composite electrolytes in lithium-polymer flexible batteries requires careful consideration of additional experimental factors. To better understand the dual role of TiO_2_ in tailoring the dynamic-mechanical and ionic properties of epoxy electrolytes, this study systematically examines the available literature. The objective of this study is to answer the following research question: how effective are TiO_2_ nanoparticles in simultaneously enhancing the dynamic-mechanical and ionic properties of epoxy-based electrolytes compared to unfilled epoxy electrolytes?

To ensure a comprehensive and systematic review, the Problem, Intervention, Comparison, and Outcome (PICO) framework from the Evidence-Based Practice (EBP)^26^ was adopted and adhered to the Preferred Reporting Items for Systematic reviews and Meta-Analyses (PRISMA) guidelines.^27^ While recent studies indicate that ceramic nanoparticles can modulate both mechanical and electrochemical behavior in polymer matrices, the extent to which dynamic-mechanical trends observed in TiO_2_-filled epoxy systems translate into ionic performance improvements remains untested. For instance, mesoporous TiO_2_ has been shown to enhance mechanical properties in epoxy formulations,^11^ whereas TiO_2_-containing polymer electrolytes exhibit conductivity gains primarily in nonepoxy matrices such as PVDF-HFP/PI.^12^ This asymmetry illustrates that mechanistic parallels across systems remain indirect. Accordingly, this study adopts an epoxy-centered perspective for dynamic-mechanical evidence, while electrolyte studies are used solely as contextual comparators. Any cross-domain interpretation is therefore exploratory and constrained by the absence of studies reporting both mechanical and electrochemical data for the same epoxy-TiO_2_ formulations. Consequently, this study does not aim to propose new structure–property relationships but rather to clarify the evidence gaps that currently prevent them from being established.

## 2 Methods

This systematic review followed the Population, Intervention, Comparison, and Outcome (PICO) index of evidence-based practice (EBP)^26^ and the Preferred Reporting Items for Systematic Reviews and Meta-Analyses (PRISMA) statement.^27^

### 2.1 Study Eligibility Criteria

Table 1 summarizes the key elements of the PICO index that form the basis for developing our research question.

**Table 1:** Summary and Description of the PICO Elements

PICO		
acronym	definition	description
P	problem	simultaneous improvement in dynamic-mechanical properties and ionic conductivity of epoxy electrolytes
I	intervention	addition of TiO_2_ nanoparticles (experimental and computer simulation studies have been considered)
C	comparator	unfilled epoxy resins compared to TiO_2_-filled epoxy resins; unfilled epoxy electrolytes compared to TiO_2_-filled epoxy electrolytes
O	outcome	TiO_2_ content, TiO_2_ size, dynamic-mechanical properties, electrochemical properties

The PICO-based research question guiding this review was: “How effective are TiO_2_ nanoparticles in simultaneously enhancing the dynamic-mechanical and ionic properties of epoxy-based electrolytes compared to unfilled epoxy electrolytes?” This question does not presuppose that these mechanical and electrochemical bodies of evidence are directly mappable, and no inclusion criteria required studies to report both outcomes for the same epoxy-based formulation; therefore, the feasibility of identifying cross-domain relationships depended entirely on the structure of the existing literature. In addition to specifying the PICO items (Table 1), the characteristics of this report were described. These include all search strategies, study records, outcomes and prioritization, risk of bias assessment, and data synthesis.

### 2.2 Search Strategy

This systematic review used the ScienceDirect, Scopus, Web of Science, and Springer databases to address the research question guided by the problem, intervention, comparator, and outcome elements of the PICO framework. Six general keywords related to the PICO elements were identified, and logical search strings were created and adapted to the syntax of each database. To ensure that each database search strategy retrieved a high proportion of eligible studies, three searches were conducted per electronic database: two searches limited to evidence on unfilled epoxy resins vs TiO_2_-filled epoxy resins and one limited to unfilled epoxy electrolytes vs TiO_2_-filled epoxy electrolytes. Documentation of the planned information sources, including the dates of coverage, is provided in Table 2. The ScienceDirect strategy was the only one listed for all three searches because no eligible studies were found for other databases on TiO_2_-free and nanocomposite electrolytes.

**Table 2:** Summary of the Sources of Information and the Search Strategy for the 2014–2024 Theme

database	search date	search string	number of studies identified				
			results	preselected	excluded	eligible	in duplicate
ScienceDirect	Apr. 24, 2024	(“epoxy resin” OR epoxy OR “epoxy nanocomposite”) AND (nanoparticle OR particle) AND (“dynamic?mechanical analys^a^” OR DMA OR “dynamic?mechanical behavior”) AND (simulation OR mode?ing)	894	1	893	1	-
	Apr. 25, 2024	“epoxy resin” AND TiO_2_ AND (nanoparticle OR particle) AND (dynamic?mechanical analys^a^ OR DMA OR “dynamic?mechanical behavior”)	59	8	51	7	-
	Oct. 11, 2024	nanocomposite AND ″dynamic mechanical″ AND ″electrochemical″	428	28	400	1^a^	-
Scopus	May 07, 2024	(“epoxy resin” OR epoxy OR “epoxy nanocomposite”) AND (nanoparticle OR particle) AND (“dynamic?mechanical analys^a^” OR DMA OR “dynamic?mechanical behavior”) AND (simulation OR mode?ing)	17	2	15	1	-
	May 07, 2024	“epoxy resin” AND TiO_2_ AND (nanoparticle OR particle) AND (“dynamic?mechanical analys^a^” OR DMA OR “dynamic?mechanical behavior”)	2	2	1	1	1
Web of Science	May 07, 2024	(“epoxy resin” OR epoxy OR “epoxy nanocomposite”) AND (nanoparticle OR particle) AND (“dynamic?mechanical analys^a^” OR DMA OR “dynamic?mechanical behavior”) AND (simulation OR mode?ing)	12	3	9	1	1
	May 08, 2024	“epoxy resin” AND TiO_2_ AND (nanoparticle OR particle) AND (“dynamic?mechanical analys^a^” OR DMA OR “dynamic?mechanical behavior”)	5	3	2	2	1
Springer	May 08, 2024	“epoxy resin” AND TiO_2_ AND (nanoparticle OR particle) AND (“dynamic?mechanical analys^a^” OR DMA OR “dynamic?mechanical behavior”)	61	6	52	5	-
	May 09, 2024	“epoxy resin” AND TiO_2_ AND (nanoparticles OR filler) AND (DMA OR “dynamic?mechanical properties”)	77	8	69	7	2

a Using the reference list of this Record, we identified four works that also appeared to meet our eligibility criteria.

### 2.3 Study Records

The results of the literature search were uploaded to a standardized form that was created specifically for this study. The following data were extracted and recorded: the surname of the last author, the year of publication, the location, the objective of each eligible study, the sample (type and size of nanoparticles/polymer matrix), the dynamic-mechanical analysis protocol, and the key findings of each eligible study.

Prior to this data collection process, we independently screened the titles and abstracts returned by each search against the following eligibility criteria: (1) full-text studies from indexed journals; (2) full-text studies published in English; (3) full-text studies published from 2014 to 2024; (4) full-text studies related to dynamic-mechanical characterization of epoxy-TiO_2_ nanocomposites; and (5) full-text studies related to electrochemical characterization of epoxy-TiO_2_ nanocomposite electrolytes.

The full text of each study that appeared to meet these criteria was reviewed to determine whether the study was eligible for the data-collection process. Since duplicate studies were identified during the screening process, these studies were excluded after reading the titles and abstracts. When necessary, inconsistencies and disagreements were resolved by discussion, and one of two arbitrators (G.L.C. or G.Z.) adjudicated unresolved disagreements.

### 2.4 Assessment of Study Quality and Risk of Bias

No judgments were made about the domain level and the overall risk of bias in the eligible studies nor were sensitivity analyses carried out to assess the robustness of the compiled outcomes. As a result of the absence of a risk of bias analysis, statistical confidence in the body of evidence was also not assessed (as explained in the next section).

### 2.5 Outcomes and Prioritization

The primary outcomes were the empirical values of Young’s modulus, loss modulus, mechanical damping, glass-transition temperature (dynamic-mechanical parameters), and ionic conductivity (electrochemical parameter). Furthermore, it was decided a priori to subdivide the primary outcomes in order to identify the influential factors in the examination of the complex relationship between the properties of nanocomposites and semisolid electrolytes properties, as follows:

1. DMA responses of epoxy-TiO_2_ nanocomposites as the review end points were given preference.
2. DMA and electrochemical responses of epoxy-TiO_2_ electrolytes as the review end points were given preference.

When necessary, data were approximated from the figures in the included studies. All percentage changes in each parameter were calculated by comparing the results of the unfilled and particle-filled polymers. Additional results (secondary outcomes) were descriptions of the underlying mechanism that explain the simultaneous improvement in dynamic-mechanical properties and ionic conductivity of epoxy electrolytes by the addition of nanoparticles. These have been summarized and presented directly in the text as part of the discussion in the next section.

### 2.6 Data Synthesis

No meta-analyses were performed (as explained in the next section). Instead, a systematic narrative synthesis was provided with information presented in the text and tables to summarize and explain the findings of the eligible studies.

Although meta-analyses were not performed, possible sources of between-study heterogeneity were explored using regression analyses. The consistency of results across studies by using Pearson’s *r* test for primary outcomes was analyzed (e.g., the data from two dynamic-mechanical variables was used, such as Tan δ and *T*_g_, to test the strength of the correlation between them) and a *p*-value < 0.05 was considered significant.

## 3 Results and Discussion

In this study, 1555 articles were found through database searching, as depicted in the flowchart in Figure 2.^28^ Among these, 26 articles appeared to meet the eligibility criteria. In this context, 11 studies^22,29–37^ were included for DMA responses of epoxy-TiO_2_ nanocomposites as the end points and one^38^ included for DMA and electrochemical responses of polymer electrolytes. Four other studies^12,39–41^ were found through citation searching in ref^38^ and included for DMA and electrochemical responses of polymer electrolytes. Refs 12,39–41 were included in a summary of findings even though these were not fully compliant with the “dynamic-mechanical characterization”, “electrochemical characterization” and “epoxy-TiO_2_” categories (as discussed later in this section). In addition, occasional references to SiO_2_ and ZrO_2_ were retained only for contextual comparison. These oxides are commonly used ceramic fillers in polymer electrolytes and structural nanocomposites, and their mention helps situate the viscoelastic and electrochemical behavior observed for TiO_2_ within broader polymer-oxide trends. Importantly, these references do not expand the scope of the review beyond TiO_2_-containing systems.

**Figure 2:**
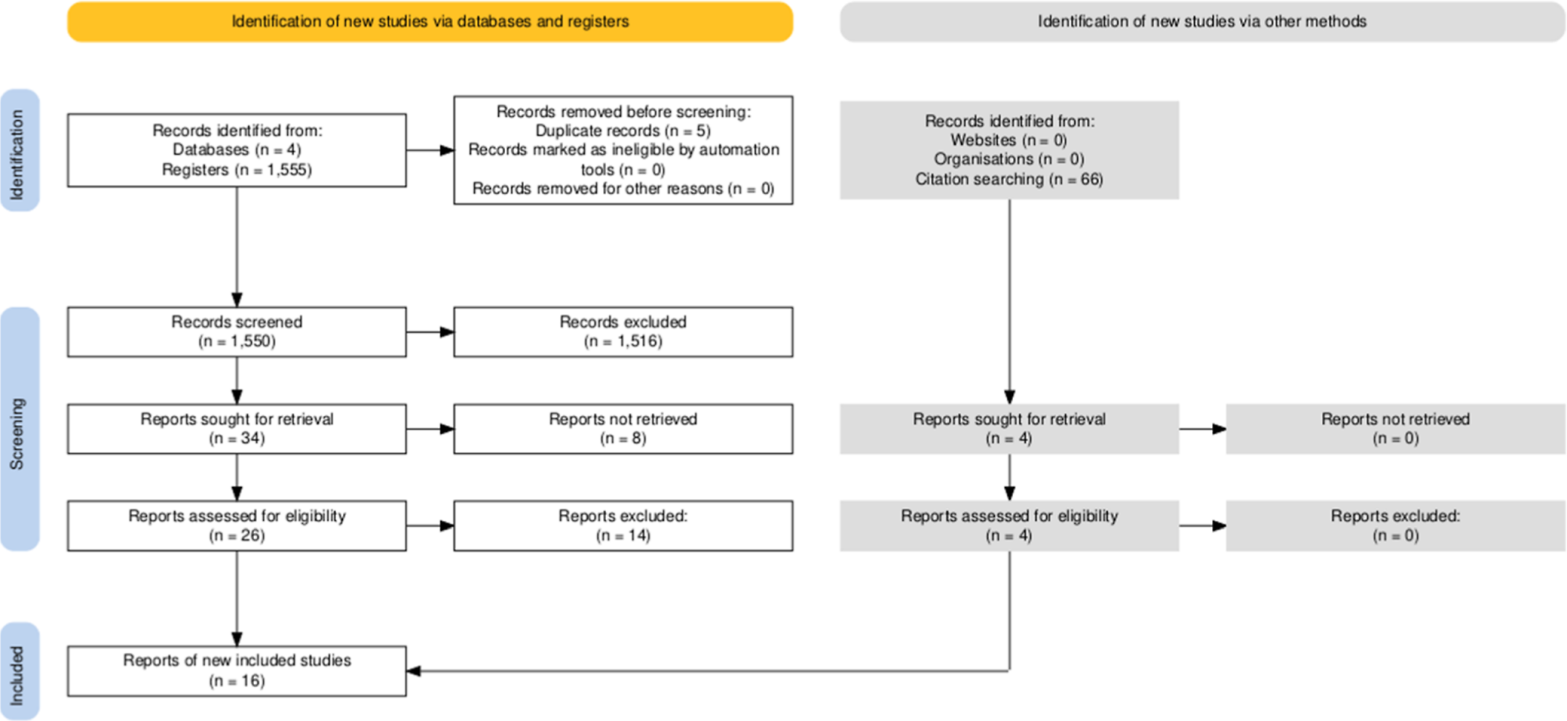
Flowchart of the studies selected for the systematic review.^28^

Of the 26 articles, other 14 records^42–55^ appeared to meet the eligibility criteria but were excluded. Among these, refs 52–55 did not comply with the criteria of full-text studies published from 2014 to 2024 and refs 42–51 were not in line with the criteria of full-text studies related to dynamic-mechanical characterization of epoxy-TiO_2_ nanocomposites.

On the basis of these, discussion on the viscoelastic characteristics of epoxy-TiO_2_ nanocomposite polymers are presented in the next section. Then, we will consider the viscoelastic characteristics of the nanocomposites in the semisolid electrolytes, compare the dynamic-mechanical properties of epoxy-TiO_2_ alone with those of the polymer electrolytes, and assess the relationships between nanocomposites and semisolid electrolytes.

### 3.1 Viscoelastic Characteristics of Epoxy-TiO_2_ Nanocomposites

The primary objective of our analysis of the viscoelastic properties of the nanocomposite matrix was to synthesize and integrate data from the included studies. The main quantitative findings, summarized in Table 3, highlight changes in storage modulus (*E*′), glass-transition temperature (*T*_g_), and damping coefficient (Tan δ) relative to particle size and weight content, providing an initial understanding of the dynamic-mechanical tailoring of the matrix.

**Table 3:** Quantitative Data Extracted from Included Studies

Author et al., (year) (ref.)	TiO_2_ nanoparticles		dynamic-–mechanical properties of epoxy					
	size (nm)	content (wt %)	*E*’ (MPa)	Δ*E*’ (%)^a^	*T*_g_ (°C)	Δ*T*_g_ (%)^a^	Tan δ (a.u.)	ΔTan δ(%)^a^
Siami Araghi et al., (2023)^29^	10–30	n/a	n/a	n/a	n/a			
Hiremath et al., (2023)^25^	21	0	n/a	107	–	0.108	–	
		0.5		112	+4.7	0.140	+30	
		1.0		118	+10	0.084	–22	
		1.5		113	+5.6	0.107	–0.9	
		2.0		110	+2.8	0.139	+29	
		2.5		108	+0.9	0.277	+156	
Roy et al., (2023)^30^	30–40	n/a	n/a	n/a	n/a			
Zahrouni et al., (2023)^31^	n/a	5.0	n/a	n/a	n/a			
Esposti et al., (2022)^32^	n/a	0	0.46	–	n/a	n/a		
		1.0	0.87	+89				
		3.0	1.38	+200				
		5.0	2.18	+374				
Bakar et al., (2021)^22^	21	0	n/a	n/a	n/a	n/a	n/a	
		0.5		78.5		0.675		
		1.25		76.5		0.66		
		1.5		77.0		0.60		
		3.75		78.0		0.58		
Malekshahinezhad et al., (2020)^33^	30	0	n/a	n/a	n/a			
		5.0						
Singh et al., (2017)^34^	32	0	175	–	60.78	–	0.832	–
		2.0	183	+4.6	66.33	+9	0.782	–6
		4.0	205	+17	69.59	+14	0.792	–5
		6.0	208	+19	72.34	+19	0.753	–9
		8.0	195	+11	70.38	+16	0.788	–5
Wang et al., (2017)^35^	21	0	n/a	n/a	n/a			
		1.0						
		2.5						
		5.0						
		7.5						
Kumar et al., (2016)^36^	30–40	0	500	–	174.6	–	0.56	–
		5.0	580	+16	177.4	+1.6	0.55	–2
		10.0	640	+28	182.4	+4.5	0.57	+2
		15.0	460	–8	169.4	–3.0	0.49	–12
Salehi et al., (2016)^37^	10–25	0	n/a	–	n/a	n/a		
		0.25 v %		+5%				
		0.5 v %		+9%				
		1.0 v %		+14%				

a Percentage changes (Δ %) refer only to the corresponding TiO_2_-free epoxy matrix.

Overall, the addition of TiO_2_ nanoparticles increased both E′ and *T*_g_ (Table 3). By comparing the available evidence in included study reports, the largest enhancements in storage modulus (+28%) and glass-transition temperature (+19%) were observed with a high particle content (10 and 6 wt %, respectively) of large particles (mean diameters: 35 and 32 nm, respectively). These results might indicate that stiffener polymers, typically those filled with larger particles, exhibit reduced deformation compared to matrices filled with smaller particles. While particle size and weight content are known to influence polymer-filler interfacial area, chain mobility, and the resulting viscoelastic response, the available epoxy-TiO_2_ studies did not report these parameters in a manner that would allow systematic comparison under matched DMA conditions. Therefore, although detailed discussion of such effects exist in the broader literature,^16^ these variables could not be quantitatively evaluated within this review and are addressed here only qualitatively.

Despite the very enlightening discussion in^16^, the aforementioned paper does not study properties concerning polymer design for application in structural batteries. This is addressed with our systematic review, but the review is dedicated to electrolyte materials for semisolid batteries. However, the discussion that follows was narrowed down to damping coefficient (Tan δ), which is critical for understanding energy dissipation through molecular rearrangements and a key factor in designing polymer matrices for structural electrolytes. At the same time, the specific case of Tan δ vs *T*_g_ to highlight changes in the flexibility of polymer chains was examined, which seems to favor *T*_g_. Figure 3 depicts the Pearson’s r correlation matrix for the primary outcomes in Table 3.

**Figure 3:**
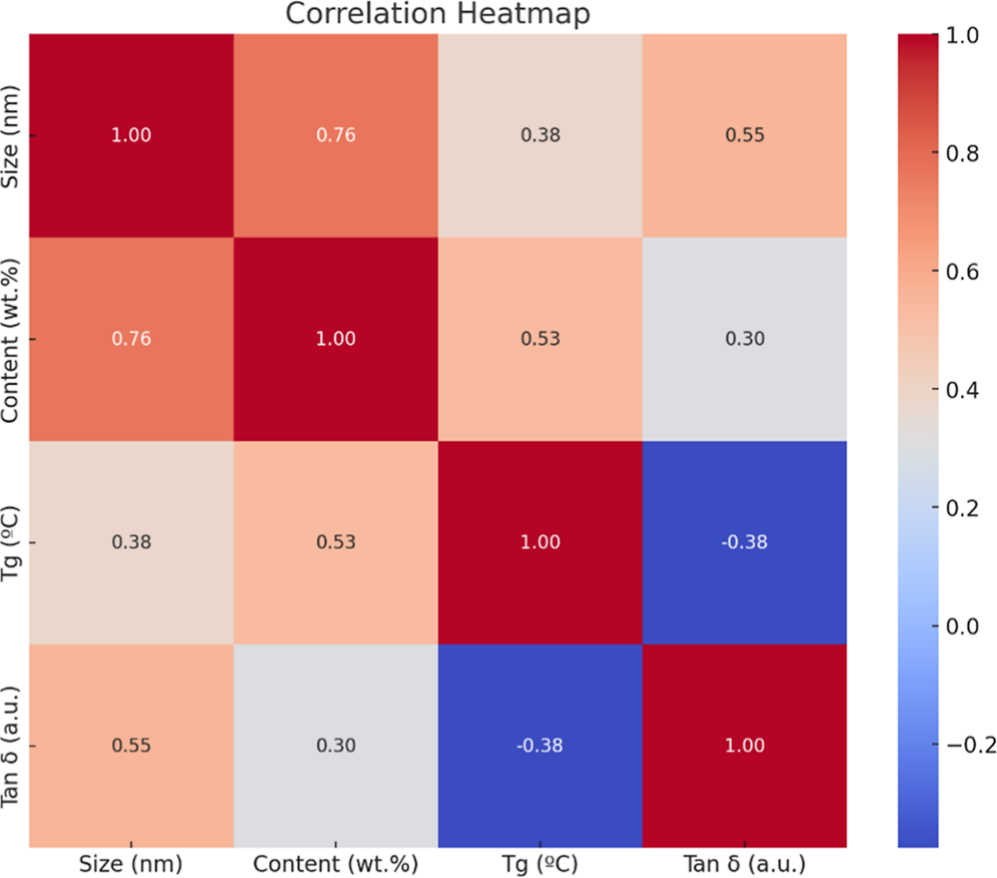
Pearson’s *r* correlation heatmap matrix between structural and dynamic-mechanical parameters for epoxy-TiO_2_ nanocomposites.

In the case of Tan δ and *T*_g_ end points, the *r* of −0.38 represents an indirect and weak correlation with no statistical significance (*p* > 0.05). The coefficient of determination (*R* ^2^ = *r* ^2^) value of 0.14 means that the probability of predicting Tan δ based on *T*_g_ is 14%. Accordingly, the *p*-value of 0.15 reassures us that there is no statistically significant correlation between Tan δ and *T*_g_ at the 5% significance level. Figure 4 complements this analysis by showing the diversity trend in Tan δ vs *T*_g_ outcomes. Possible sources leading to the observed heterogeneity may have differences in formulations and conditions of the experimental characterization. The studies were examined in detail to consider whether to compare and highlight the trade-off between damping behavior and mechanical load capacity.

**Figure 4:**
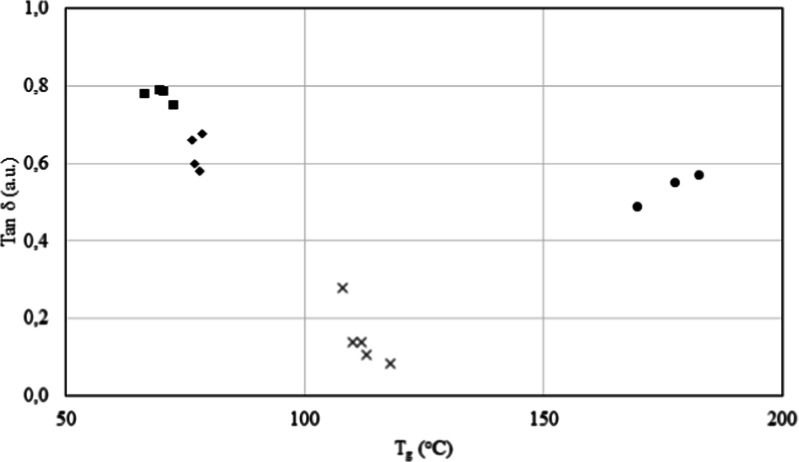
loss factor (Tan δ) and corresponding glass-transition temperature (*T*_g_) for various epoxy-TiO_2_ nanocomposites: 32 nm TiO_2_ nanocomposites from Singh et al.,^34^ (square symbol), 21 nm TiO_2_ nanocomposites from Bakar et al.,^22^ (diamond symbol), 21 nm TiO_2_ nanocomposites from Hiremath et al.,^25^ (x symbol), and 30–40 nm TiO_2_ nanocomposites from Kumar et al.,^36^ (circle symbol). Data were compiled and integrated from the included studies.

Table 4 summarizes the differences in formulations and conditions of the experimental characterization (such as measurement protocols, specimen type, and specimen preparation method for dynamic-mechanical analyses) of the epoxy-based nanocomposites in the studies used to design Figure 4.

**Table 4:** Summary of the Main Nanocomposite Characteristics and Sample Specifications for DMA

Author et al., (year) (ref.)	formulation specs					
	reinforcing phase composition	TiO_2_ nanoparticles characteristics		testing conditions		
		size (nm)	content (wt %)	measurement protocol	sample specs	
					specimen type	preparation method
Hiremath et al., (2023)^25^	TiO_2_ Glass fiber	21	0.5–2.5	n/a.	laminates: 300 × 150 × 4 mm.	compression molding
Bakar et al., (2021)^22^	TiO_2_ MMT K10 Aramide	21	0.5–3.8	Mode: compression. Deformation: <0.6 mm. Frequency: 10 Hz. Time: 20 h. Temperature: 20–140 °C. Heating rate: 0.1 °C/min	plates	poured into molds and cured at room temperature
Singh et al., (2017)^34^	TiO_2_	32	2.0–8.0	Mode: three-point bending. Amplitude: 0.02 mm. Frequency: 1 Hz. Temperature: 25–100 °C. Heating rate: 2 °C/min	test specimens: 56 × 12 × 3 mm	poured into molds and cured in oven at 40 °C
Kumar et al., (2016)^36^	TiO_2_	30–40	5.0–15.0	Mode: single-cantilever. Frequency: 1 Hz. Temperature: 40–250 °C. Heating rate: 2 °C/min	test specimens: 25 × 8 × 2.5 mm	poured into metal with Teflon molds and cured in oven at 120 °C

We find that pooling data from multiple studies is challenging due to variability in the formulation and characterization of epoxy-TiO_2_ nanocomposites. The dynamic-mechanical properties are strongly influenced by these factors, underscoring the need for critical evaluation of nanostructuring technology and experimental testing to optimize property–structure relationships toward automotive application. In practice, our ability to assess the risk of bias across studies was limited, and consequently, meta-analyses could not be performed.

Because the studies by Singh et al.^34^ and Kumar et al.^36^ exhibit sufficiently aligned epoxy-TiO_2_ formulations and DMA testing conditions, they were treated as a coherent subgroup for exploratory quantitative comparison. To avoid bias from multiple statistical contrasts, their outcomes were combined and analyzed jointly. Accordingly, the correlation analysis (Figure 4) was conducted exclusively on these two studies, which represent the most internally consistent DMA protocols within the evidence set; all other entries in Tables 3 and 4 are synthesized descriptively and were not used for quantitative inference. In this subgroup, the correlation between Tan δ and *T*_g_ yielded a Pearson’s *r* of −0.97, indicating an indirect and very strong-to-perfect linear association: higher *T*_g_ values are associated with lower tan δ. The coefficient of determination (*R* ^2^ = 0.94) indicates that approximately 94% of the variability in tan δ can be explained by Tg, and the *p*-value (0.0004) confirms that this negative correlation is statistically significant under conventional thresholds (α = 0.05). Collectively, these results illustrate a clear trade-off between damping behavior and mechanical load-bearing capacity, as depicted in Figure 5.

**Figure 5:**
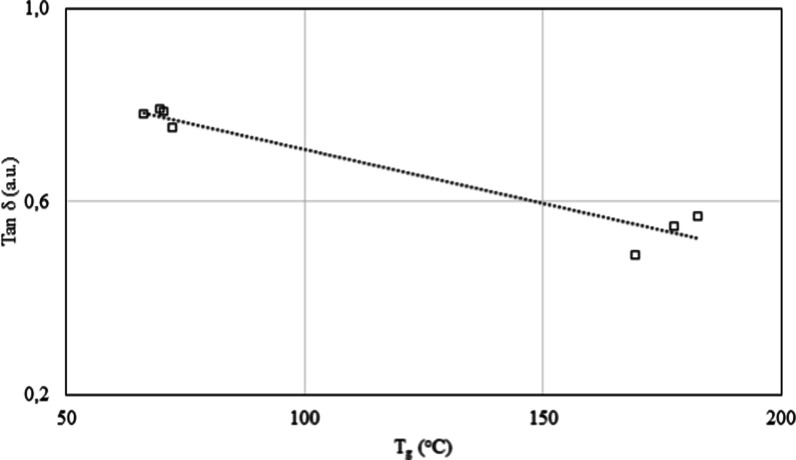
Tan δ versus *T*_g_ for epoxy-TiO_2_ nanocomposites based on Singh et al.^34^ (32 nm TiO_2_) and Kumar et al.^36^ (30–40 nm TiO_2_), the two studies with DMA protocols sufficiently aligned to enable an exploratory statistical correlation. Remaining data sets in Tables 3 and 4 were not included in the analysis.

The fact that the low number of epoxy-TiO_2_ samples^11^ used in this evaluation reduces the accuracy of the analysis significantly is of equal importance. Recognizing this limitation, a structure–property trend analysis was performed as an auxiliary tool for comparison, and it should be noted that the accuracy of this analysis may be low. Although this systematic review addresses deficiencies in risk of bias assessment, it might appear that higher TiO_2_ concentrations (∼6–10 wt %), at an almost similar particle size of 30–40 nm, are more suitable when high modulus values is a priority (compared with the unfilled polymer). Conversely, if energy dissipation is prioritized over mechanical load-bearing capacity, lower TiO_2_ concentrations (∼2–6 wt %) may be more suitable. Thus, when specifically considering the particle influence between Tan δ and *T*_g_, the particle weight content, at an almost fixed particle size of 30–40 nm, is a critical variable for evaluation.

These findings might allow for the estimation of dynamic-mechanical properties of epoxy-TiO_2_ nanocomposites and may provide guidance in selecting an appropriate nanostructured matrix for application in polymer electrolytes. However, this systematic review is not sensitive enough to discern clear differences of downscaling together the particle size and the particle weight content on the relationship between Tan δ and *T*_g_ since we did not include the results of Hiremath et al.^25^ and Bakar et al.^22^ in the regression analysis.

In summary, the dynamic-mechanical properties of epoxy-TiO_2_ nanocomposites were systematically examined in this section, yielding insights consistent with previous research.^16^ The wide range of epoxy-TiO_2_ nanocomposites available to meet the diverse technical requirements of battery separator films necessitates the careful selection of materials. Tools such as Figure 5 may provide valuable guidance for identifying optimal combinations of TiO_2_ nanoparticles and epoxy resins for specific applications in lithium-polymer batteries. In the subsequent section, we will consider the viscoelastic characteristics of the nanocomposites in the semisolid electrolytes, compare the dynamic-mechanical properties of the epoxy electrolytes with those of the matrices, and explore the relationships between nanocomposites and semisolid electrolytes to assess whether TiO_2_ nanoparticles are effective in simultaneously enhancing the dynamic-mechanical and ionic properties of epoxy-based electrolytes.

Whether the mechanical trends synthesized here could translate into electrochemical performance enhancements in epoxy-based electrolytes depend on the existence of studies reporting both domains for the same formulation. As demonstrated in Section 3.2, such paired evidence is currently absent.

### 3.2 Viscoelastic and Electrochemical Characteristics of Nanocomposite Electrolytes

To evaluate the viscoelastic and electrochemical characteristics of the lithium-polymer electrolytes, data from the included studies^12,38–41^ were synthesized. The primary findings are presented in Table 5. Despite lacking sample designs and dynamic-mechanical data, refs 12,38–41 were considered to assess due to the low number of eligible studies retrieved by this database search strategy. However, although the review was originally designed to investigate a possible link between the dynamic-mechanical behavior of epoxy/TiO_2_ nanocomposites and the electrochemical performance of semisolid epoxy-based electrolytes, this connection could not be established because no included studies simultaneously reported both mechanical and electrochemical metrics for the same epoxy formulations. Furthermore, all conductivity enhancements identified in polymer–particle electrolytes were observed exclusively in nonepoxy matrices (e.g., PVDF-HFP, PMMA, PI, PEO), meaning that these findings do not constitute evidence that TiO_2_-induced mechanical improvements in epoxy systems translate into electrochemical gains.

**Table 5:** Viscoelastic and Electrochemical Data Extracted from Included Studies

Author et al., (year) (ref.)	nanoparticles			polymer type	dynamic-mechanical properties				Li–Po conductivity	
	type	size (nm)	content (wt %)		*E*′ (MPa)	Δ*E*′ (%)^a^	*T*_g_ (°C)	Tan δ (a.u.)	σ (mS·cm^–1^)	Δσ (%)^b^
Jamalpour et al., (2021)^38^	SiO_2_	5–15	0	PVDF	24.6	–	n/a.	n/a.	1.17	–
			10.0		54.6	+122			3.10	+165
Zhai et al., (2016)^39^	TiO_2_	10	0	PVDF-HFP	n/a.	n/a.	n/a.	0.5	–	
			10.0					2.0	+300	
Chen et al., (2015)^12^	TiO_2_	20–30	0	polyimide/PVDF-HFP	n/a.	n/a.	n/a.	1.18	–	
			2.0					2.36	+100	
Song et al., (2015)^40^	TiO_2_	n/a.	0	PMMA/PVDF-HFP	n/a.	n/a.	n/a.	1.35	–	
			2.0					1.62	+20	
			5.0					2.49	+84	
			7.0					1.93	+43	
TianKhoon et al., (2015)^41^	TiO_2_	8–15	0	PMMA/PVDF-HFP	n/a.	n/a.	n/a.	n/a	–	
			6.0					7 × 10^–4^	–	

a Percentage changes (Δ %) refer only to the corresponding nanoparticle-free polymer matrix.

b Percentage changes (Δ %) refer only to the corresponding nanoparticle-free electrolyte.

Nevertheless, the results in Table 5 demonstrate a clear enhancement in conductivity within the nanocomposite polymer electrolytes. Additionally, studies by Jamalpour et al.^38^ and Chen et al.^12^ provide valuable insights through mechanical and electrochemical measurements.

The considerable enhancement in modulus, along with a 165% improvement in ionic conductivity, demonstrated by Jamalpour et al.,^38^ and the significant increase in Young’s modulus, as revealed by the stress–strain analysis in ref^12^ along with a 100% improvement in ionic conductivity, suggest that improvements in the polymer matrix’s conductivity are accompanied by enhanced mechanical properties of the semisolid electrolyte when transitioning from nanoparticle-free to nanocomposite electrolytes. Based on the summary of the secondary outcomes (Table 6) and supported by the insightful discussion in ref^56^, it was envisaged that nanometer-sized ceramics may act as nanostructuring agents for polymers, beyond fillers, that rearrange the polymer structure at the chain-particle interface, leading to a strengthened structure that favors preferential Li^+^ transport pathways. However, conclusions based on the low number of studies should be interpreted cautiously and may not constitute strong evidence.

**Table 6:** Qualitative Data from the Included Studies

Author et al., (year) (ref.)	additional remarks
Jamalpour et al., (2021)^38^	The reduced crystallinity of the membranes suggested that the polymer–particle interaction limited the crystallization of the polymer. The distinctive structure helped to absorb electrolyte solution, thus improving the ionic conductivity of the membrane. In addition, the increase in E′ confirmed the reinforcing effect of the nanoparticles in the membranes.
Zhai et al., (2016)^39^	The activation energy was correlated with the crystallinity of the electrolyte and the formation of a new lithium-ion conducting path, since low crystallinity corresponds to high mobility of the polymer chains and the new conducting path transmits more lithium ions.
Chen et al., (2015)^12^	The higher ionic conductivities of the polymer–nanoparticle electrolytes were mainly attributed to higher liquid electrolyte uptake and better liquid electrolyte wettability, which contributed to the formation of more channels allowing more lithium ions to migrate through the polymer electrolyte. Furthermore, the excellent affinity between the embedded liquid electrolyte and the polar surface groups of the nanoparticles also contributed to the improved ionic conductivities of the modified polymer electrolytes. In addition, the covalent attachment to the polymer chains could enhance the interaction between the nanoparticles and the polymer matrix, which served to further improve the mechanical properties of the membranes.
Song et al., (2015)^40^	It was found that after the introduction of nanoparticles, the room temperature ionic conductivity of the polymer electrolyte was significantly enhanced. The significant improvement in ionic conductivity was attributed to the reticular porous morphology and suitable pore size of the polymer electrolyte, which also enhanced the electrolyte uptake and transfer of lithium ions.
TianKhoon et al., (2015)^41^	The nanosized ceramic surface groups were expected to favor interactions (hydrogen bonding) with both lithium ions and polymer segments, leading to an increase in lithium-based salt dissociation in the local polymer amorphous phase, which could explain the ionic conductivity results obtained.

As summarized in Table 6, most mechanistic insights into nanoparticle-induced structural rearrangements such as Lewis acid surface interactions, segmental mobility modulation, and chain-particle interfacial restructuring originate from nonepoxy polymer–particle electrolyte systems (e.g., PVDF-HFP, PMMA, PI). These hypotheses are consistent with the broader discussion provided in ref^56^ and help rationalize how nanosized ceramics may act as nanostructuring agents that facilitate Li^+^ transport. However, because none of the included studies report paired mechanical and electrochemical measurements for the same epoxy-based formulation, such mechanisms remain hypothesis generating rather than evidence-supported for epoxy systems. This reinforces that the interpretations drawn here cannot be generalized beyond the nonepoxy matrices represented in Table 6 and should be viewed as contextual rather than confirmatory.

Taken together, the additional descriptors compiled in Table 7 reinforce that most of the relevant electrochemical information, lithium-ion transference number (tLi^+^), electrochemical stability window (ESW), interfacial resistance evolution, and full-cell cycling behavior, is derived exclusively from nonepoxy polymer matrices. The literature also does not report structural-mechanical indicators such as strain-to-failure (in tension and compression) or cure shrinkage, which would be necessary to accurately predict internal stress states. As a result, these data sets function primarily as contextual comparators rather than as evidence capable of validating a direct mechanical-electrochemical translation for epoxy-based electrolytes. This underscores the structural limitations of the available literature and highlights the need for future epoxy-oriented studies to adopt integrated mechanical and electrochemical reporting.

**Table 7:** Expanded Electrochemical Descriptors (tLi^+^, ESW, Interfacial Resistance, and Cycling Metrics) Extracted from Nonepoxy Nanoparticle-Filled Polymer Electrolytes Included as Contextual Comparators in This Review

Author (year) (ref)	polymer matrix	Filler	tLi^+^	ESW	*R*_int_ evolution	full cell
Jamalpour et al. (2021)^38^	PVDF	SiO_2_	n/a	∼4.8 V vs Li^+^/Li (RT, LSV)	580 Ω; no time-evolution (RT, EIS)	LMO∥Li: ∼ 88% capacity retention @ 50 cycles, 0.2 C; CE ∼ 90%
Zhai et al. (2016)^39^	PVDF-HFP	TiO_2_	0.56 (30 °C, EIS)	∼6 V vs Li^+^/Li (30 °C, LSV)	∼150 Ω → ∼ 300 Ω (8 days) → ∼ 400 Ω (stable) (30 °C, EIS)	LFP∥Li: ∼ 97.6% capacity retention @ 50 cycles, 0.5 C
Chen et al. (2015)^12^	Polyimide/PVDF-HFP	TiO_2_	n/a	∼4.5 V vs Li^+^/Li (RT, LSV)	Qualitatively lower for TiO_2_-modified electrolytes; no time-evolution (RT, EIS)	LCO∥Li: 95% capacity retention @ 50 cycles, 0.5 C
Song et al. (2015)^40^	PMMA/PVDF-HFP	TiO_2_	n/a	∼4.75 V vs Li^+^/Li (RT, LSV)	Qualitatively lower for TiO_2_-modified electrolytes; no time-evolution (RT, EIS)	LCO∥Li: 92.1% capacity retention @ 50 cycles, 0.2 C
TianKhoon et al., (2015)^41^	PMMA/PVDF-HFP	TiO_2_	n/a	n/a	n/a	Li∥Graphite: 198.5 → 301.7 mAh.g^–1^; CE: 64.2→98.6% @ 4 cycles

This asymmetry between the available mechanical evidence (epoxy-based) and electrochemical evidence (nonepoxy) underscores a structural gap in the literature and highlights the need for epoxy-based electrolytes to be evaluated using integrated dynamic-mechanical and electrochemical characterization.

At this point, it would be a digression to discuss the effects of particle size and weight content on the enhanced conductivity of the semisolid electrolytes. Therefore, a linear regression analysis was performed as an indicator for the relationship between polymer–particle structure and electrolyte-particle electrochemical performance. Figure 6 depicts the Pearson’s *r* correlation matrix for the primary outcomes in Table 5.

**Figure 6:**
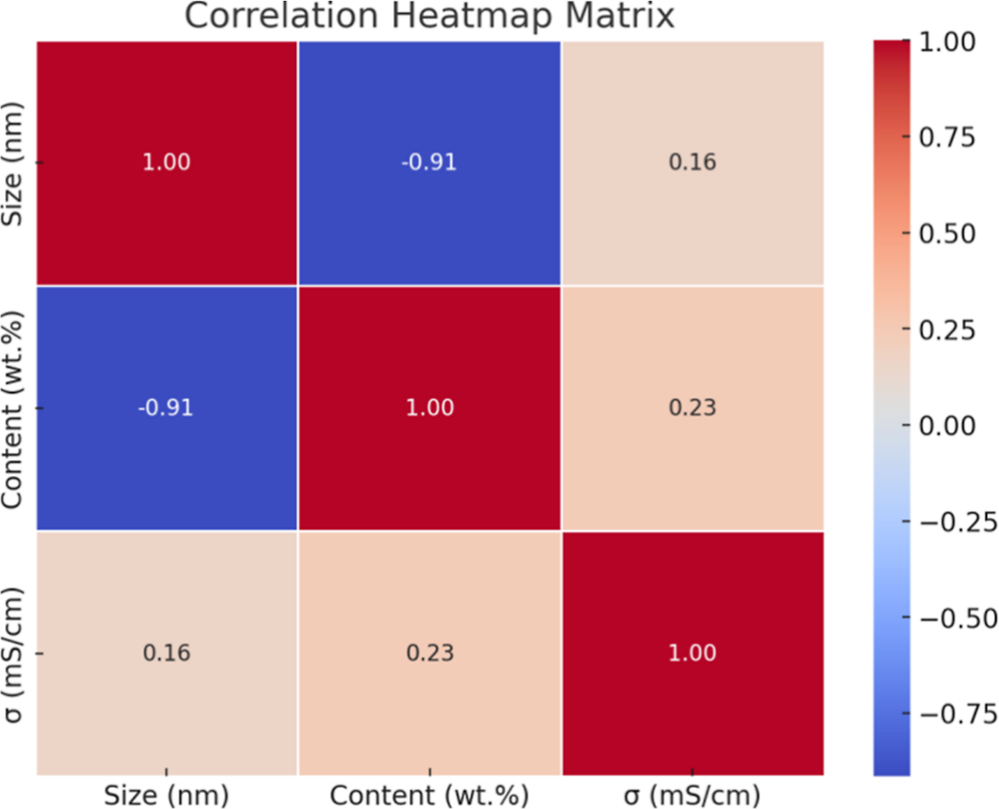
Pearson’s *r* correlation heatmap matrix between polymer–particle structural and electrolyte-particle electrochemical parameters.

In the cases of σ vs particle size and σ vs particle content, the values of *r* represent weak correlation. These results suggest that there is much work to be done to elucidate the dual role of nanoparticles in tailoring the mechanical and ionic responses of semisolid electrolytes.

In addition, no statistically significant correlation between σ and particle size (*p* = 0.84) were clearly observed because the p-value is much greater than the common significance level (e.g., 0.05). In other words, the null hypothesis could not be rejected, suggesting that any observed correlation (*r* = 0.16, i.e., a positive and weak correlation) is likely due to random chance. The Pearson correlation coefficient between σ and particle content is 0.23, indicating a positive and also weak correlation. However, the *p*-value is 0.66, which is still much higher than the common significance level of 0.05. This means there is no statistically significant evidence to suggest a meaningful correlation between these two variables. In conclusion, it is highly challenging to express the conductivity of the polymer–particle electrolytes using isolated factors (e.g., particle size and particle weight content), as summarized in Table 8.

**Table 8:** Coefficient of Determination (*R* ^2^) and Probability (*p*) Values from Linear Regression Analysis between Polymer–Particle Structural and Electrolyte-Particle Electrochemical Parameters

entry			electrolyte-particle parameter	additional remarks
			σ (mS·cm^–1^)	
polymer–particle parameter	particle size (nm)	*R* ^2^	0.03	only 3% of the variance in σ can be explained by particle size
		*p*-value	0.84	insufficient evidence to suggest a significant correlation between particle size and σ
	particle content (wt %)	*R* ^2^	0.05	only 5% of the variance in σ can be explained by particle content
		p-value	0.66	insufficient evidence to suggest a significant correlation between particle content and σ

Further complex differences in formulations and measures of ionic conductivity, as summarized in Tables 5 and 9, limit the comparison of results obtained under different conditions by different research groups. Therefore, the effects of particle size and particle weight content on the enhanced conductivity of the semisolid electrolytes could not be assessed by our systematic review.

**Table 9:** Summary of the Main Lithium-Polymer Electrolyte Characteristics and Test Specifications for Ion Conduction Measurement

	formulation specs					
Author et al., (year) (ref.)	reinforcing phase composition	nanoparticles characteristics		membrane structure	test specs for Li^+^ conduction measurement	
		size (nm)	content (wt %)		measurement protocol	additional remarks
Jamalpour et al., (2021)^38^	Functionalized SiO_2_	5–15	10	Porosity: 69%. Electrolyte uptake: 536%. Bulk resistance: 6.3 Ω	Technique: EIS. Frequency: 1 to 10^6^ Hz. Temperature: room.	Membranes (100 mm in thickness and 2.24 cm in diameter) assembled in cells with symmetrical stainless steel (SS) electrodes in an AC impedance analyzer
Zhai et al., (2016)^39^	TiO_2_	10	10	Crystallinity: 7.4%. Electrolyte uptake: n/a.. Bulk resistance: n/a.	Technique: EIS. Frequency: 0.1 to 10^6^ Hz. Temperature: 30–80 °C.	Membranes assembled in cells with SS electrodes in an AC impedance analyzer inside an Ar-filled glovebox
Chen et al., (2015)^12^	Functionalized TiO_2_	20–30	2	Porosity: >60%. Electrolyte uptake: >400%. Bulk resistance: 0.6 Ω	Technique: EIS. Frequency: 0.1 to 10^3^ Hz. AC amplitude: 5 mV. Temperature: room.	Membranes assembled in cells with SS electrodes in an AC impedance analyzer
Song et al., (2015)^40^	P25 TiO_2_	n/a	5	Porosity: high. Pore size: 100–200 nm. Electrolyte uptake: 267%. Bulk resistance: 1.9 Ω	Technique: EISFrequency: 1 to 10^3^ Hz. Temperature: n/a.	Used an electrochemical workstation, CHI660D
TianKhoon et al., (2015)^41^	ZrO_2_ TiO_2_	8–15	20 6	Porosity: n/a. Electrolyte uptake: n/a. Bulk resistance: n/a.	Technique: EIS. Frequency: 0.1 to 10^6^ Hz. AC amplitude: 100 mV. Temperature: n/a.	Sample cell consisted of two parallel SS electrodes with effective surface contact area of 2 cm^2^

The scarcity of empirical studies addressing this theme between 2014 and 2024 suggests that the field remains underdeveloped, limiting the availability of robust evidence. As a result, the potential reliability of the evidence generated within each eligible study was not examined and could not translate the findings into potential risk of bias. Further experimental research is needed to substantiate these preliminary observations and advance our understanding of the interplay between viscoelastic and electrochemical properties in nanocomposite electrolytes.

Although the dual-role hypothesis—ceramic fillers reinforcing polymer networks while simultaneously facilitating Li^+^ transport—remains mechanistically plausible, the present evidence base does not allow this concept to be evaluated within epoxy-based systems. All electrochemical improvements identified were limited to nonepoxy polymers, preventing meaningful extrapolation to epoxy electrolytes. Thus, the mechanical-electrochemical bridge that motivated this review remains an open question pending studies specifically designed to probe it.

## 4 Conclusions

In this study, a comprehensive and systematic review was conducted to assess whether TiO_2_ nanoparticles can simultaneously enhance the dynamic-mechanical properties of epoxy matrices and the ionic properties of polymer electrolytes. While the mechanical component of this question was supported by strong trends, such as the inverse correlation between tan δ and *T*_g_ observed in selected epoxy-TiO_2_ nanocomposites, the ionic component could not be directly evaluated, since none of the included electrolyte studies involved epoxy-based formulations. Accordingly, the central research question could only be addressed in part.

The review confirmed that TiO_2_ nanoparticles can successfully tailor the viscoelastic behavior of epoxy polymers, improving the stiffness and thermal stability under certain conditions. However, due to the absence of studies reporting both dynamic-mechanical and electrochemical performance for the same epoxy-based electrolyte formulations, it remains unknown whether such mechanical gains translate into electrochemical improvements in epoxy-based semisolid electrolytes.

Importantly, the lack of precise conclusions regarding the effects of TiO_2_ particle size and filler loading on ionic conductivity reflects the current state of the literature and should be interpreted as a critical evidence gap rather than as a limitation or inconclusive outcome of this review. This limitation underscores the early stage of development of epoxy electrolytes and the structural gap in the existing literature.

Taken together, this study emphasizes the need for targeted empirical studies that jointly measure dynamic-mechanical and electrochemical metrics (ionic conductivity, tLi^+^, ESW, interfacial resistance evolution, full-cell cycling) within the same epoxy-based formulations. Only through such integrated approaches will it be possible to establish whether TiO_2_ nanoparticles can concurrently strengthen epoxy matrices and enhance ionic transport, ultimately defining their viability for structurally robust, fast-charging lithium polymer flexible batteries.

In summary, this systematic review contributes to the field by demonstrating that the current literature does not yet provide paired dynamic-mechanical and electrochemical data for epoxy-TiO_2_ electrolytes and therefore cannot support direct structure–property correlations. By clarifying this evidence gap and consolidating the trends reported across heterogeneous nanocomposite systems, the study establishes the methodological and reporting benchmarks required for future studies to advance epoxy-based polymer electrolytes. These findings underscore the need for future studies that jointly report mechanical and electrochemical properties for the same epoxy-TiO_2_ formulations under standardized conditions.
